# Supplementary material for: Characterising the nationwide burden and predictors of unkept outpatient appointments in the National Health Service in England: A cohort study using a machine learning approach
Source: PLoS Med. 2021 Oct 12;18(10):e1003783. doi: 10.1371/journal.pmed.1003783 (PMC8509877; doi:10.1371/journal.pmed.1003783)
Supplement: S2 Table — (DOCX) [file pmed.1003783.s002.docx]

S2 Table

Unkept appointments rate by UK Hospital Trust

|  |  | Pre-Cleaning | | | Post-Cleaning | | | Data Quality | | |
| --- | --- | --- | --- | --- | --- | --- | --- | --- | --- | --- |
| Code | Healthcare Trust | Appts | Unkept Appts | Unkept Rate | Appts | Unkept Appts | Unkept Rate | Appts Retained | Unkept Retained | Unkept % Diff |
| R1H | Barts Health | 2,037,612 | 182,028 | 8.9% | 1,481,137 | 178,133 | 12.0% | 72.7% | 97.9% | 3.1% |
| RAL | Royal Free | 1,973,691 | 161,947 | 8.2% | 1,510,631 | 161,928 | 10.7% | 76.5% | 100.0% | 2.5% |
| RTD | Newcastle Upon Tyne | 1,868,286 | 135,519 | 7.3% | 1,423,005 | 135,404 | 9.5% | 76.2% | 99.9% | 2.3% |
| RHQ | Sheffield Teaching Hospitals | 1,804,230 | 99,455 | 5.5% | 1,419,334 | 99,383 | 7.0% | 78.7% | 99.9% | 1.5% |
| RJZ | King's College Hospital | 1,782,377 | 150,813 | 8.5% | 1,582,314 | 150,813 | 9.5% | 88.8% | 100.0% | 1.1% |
| RWE | Leicester | 1,554,443 | 79,213 | 5.1% | 1,120,643 | 79,210 | 7.1% | 72.1% | 100.0% | 2.0% |
| RTH | Oxford Radcliffe | 1,546,829 | 66,310 | 4.3% | 1,128,150 | 66,221 | 5.9% | 72.9% | 99.9% | 1.6% |
| RJ1 | Guy's and St Thomas' | 1,505,026 | 178,938 | 11.9% | 1,502,118 | 178,621 | 11.9% | 99.8% | 99.8% | 0.0% |
| RYJ | Imperial College | 1,496,958 | 122,090 | 8.2% | 1,088,337 | 122,077 | 11.2% | 72.7% | 100.0% | 3.1% |
| RR8 | Leeds Teaching Hospitals | 1,411,927 | 102,115 | 7.2% | 1,411,477 | 102,115 | 7.2% | 100.0% | 100.0% | 0.0% |
| RX1 | Nottingham University | 1,339,314 | 78,627 | 5.9% | 1,037,053 | 78,627 | 7.6% | 77.4% | 100.0% | 1.7% |
| RDU | Frimley Park | 1,321,920 | 64,894 | 4.9% | 1,109,480 | 64,859 | 5.8% | 83.9% | 99.9% | 0.9% |
| RGT | Cambridge University | 1,318,391 | 51,252 | 3.9% | 951,113 | 50,892 | 5.4% | 72.1% | 99.3% | 1.5% |
| RW3 | Central Manchester University | 1,295,565 | 126,088 | 9.7% | 1,122,231 | 126,078 | 11.2% | 86.6% | 100.0% | 1.5% |
| RCB | York | 1,278,605 | 64,484 | 5.0% | 1,055,919 | 64,356 | 6.1% | 82.6% | 99.8% | 1.1% |
| RF4 | Barking, Havering and Redbridge | 1,131,435 | 101,005 | 8.9% | 859,744 | 100,932 | 11.7% | 76.0% | 99.9% | 2.8% |
| RR1 | Heart of England | 1,130,472 | 111,371 | 9.9% | 1,130,472 | 111,371 | 9.9% | 100.0% | 100.0% | 0.0% |
| RJE | North Staffordshire | 1,092,193 | 68,845 | 6.3% | 916,666 | 68,384 | 7.5% | 83.9% | 99.3% | 1.2% |
| RM1 | Norfolk & Norwich | 1,079,270 | 42,333 | 3.9% | 814,819 | 42,253 | 5.2% | 75.5% | 99.8% | 1.3% |
| RQM | Chelsea and Westminster | 1,072,721 | 99,123 | 9.2% | 976,891 | 99,089 | 10.1% | 91.1% | 100.0% | 0.9% |
| RJ7 | St George's | 1,066,885 | 87,816 | 8.2% | 739,336 | 87,751 | 11.9% | 69.3% | 99.9% | 3.6% |
| RVR | Epsom and St Helier | 1,063,925 | 42,715 | 4.0% | 826,911 | 42,641 | 5.2% | 77.7% | 99.8% | 1.1% |
| RRK | University Hospital Birmingham | 1,056,465 | 67,236 | 6.4% | 1,055,933 | 67,163 | 6.4% | 99.9% | 99.9% | 0.0% |
| RLN | City Hospitals Sunderland | 1,038,143 | 80,618 | 7.8% | 877,865 | 80,618 | 9.2% | 84.6% | 100.0% | 1.4% |
| RX4 | Cumbria, Northumberland, Tyne and Wear | 1,028,936 | 135,011 | 13.1% | 848,977 | 125,316 | 14.8% | 82.5% | 92.8% | 1.6% |
| RA7 | University Hospitals Bristol | 1,018,456 | 55,139 | 5.4% | 761,152 | 55,139 | 7.2% | 74.7% | 100.0% | 1.8% |
| NVC | Ramsay Healthcare Operations Limited | 1,015,691 | 44,811 | 4.4% | 904,977 | 44,811 | 5.0% | 89.1% | 100.0% | 0.5% |
| RHM | Southampton | 1,013,349 | 52,274 | 5.2% | 715,776 | 52,153 | 7.3% | 70.6% | 99.8% | 2.1% |
| RVV | East Kent | 1,005,571 | 66,297 | 6.6% | 993,830 | 66,222 | 6.7% | 98.8% | 99.9% | 0.1% |
| RXK | Sandwell and West Birmingham | 1,005,149 | 79,120 | 7.9% | 900,002 | 79,120 | 8.8% | 89.5% | 100.0% | 0.9% |
| RL4 | Royal Wolverhampton | 1,003,152 | 76,910 | 7.7% | 1,002,867 | 76,882 | 7.7% | 100.0% | 100.0% | 0.0% |
| RWD | United Lincolnshire | 994,956 | 62,523 | 6.3% | 739,943 | 62,523 | 8.4% | 74.4% | 100.0% | 2.2% |
| RXH | Brighton and Sussex | 957,584 | 59,213 | 6.2% | 714,341 | 59,166 | 8.3% | 74.6% | 99.9% | 2.1% |
| RTG | Derby Hospitals | 948,608 | 52,904 | 5.6% | 822,916 | 52,823 | 6.4% | 86.7% | 99.8% | 0.8% |
| RQ6 | Royal Liverpool and Broadgreen | 925,819 | 86,595 | 9.4% | 821,153 | 86,509 | 10.5% | 88.7% | 99.9% | 1.2% |
| RTR | South Tees | 906,046 | 72,722 | 8.0% | 904,658 | 72,564 | 8.0% | 99.8% | 99.8% | 0.0% |
| RWH | East and North Hertfordshire | 877,827 | 56,493 | 6.4% | 703,478 | 56,483 | 8.0% | 80.1% | 100.0% | 1.6% |
| RHU | Portsmouth | 864,396 | 40,052 | 4.6% | 846,654 | 40,052 | 4.7% | 97.9% | 100.0% | 0.1% |
| RWP | Worcester Acute | 847,083 | 50,655 | 6.0% | 828,471 | 50,632 | 6.1% | 97.8% | 100.0% | 0.1% |
| RKB | Coventry and Warwickshire | 846,122 | 56,669 | 6.7% | 843,376 | 56,662 | 6.7% | 99.7% | 100.0% | 0.0% |
| RTE | Gloucestershire Hospitals | 843,180 | 56,407 | 6.7% | 841,635 | 56,303 | 6.7% | 99.8% | 99.8% | 0.0% |
| NDA | Norfolk Deaf Association | 839,975 | 31,175 | 3.7% | 768,820 | 31,085 | 4.0% | 91.5% | 99.7% | 0.3% |
| R1K | London North West University | 799,443 | 116,126 | 14.5% | 799,181 | 116,077 | 14.5% | 100.0% | 100.0% | 0.0% |
| RJ2 | Lewisham and Greenwich | 787,762 | 97,911 | 12.4% | 722,687 | 97,895 | 13.5% | 91.7% | 100.0% | 1.1% |
| NT4 | BMI The London Independent | 770,370 | 27,402 | 3.6% | 709,072 | 27,397 | 3.9% | 92.0% | 100.0% | 0.3% |
| RW6 | Pennine Acute | 767,012 | 80,001 | 10.4% | 763,031 | 79,990 | 10.5% | 99.5% | 100.0% | 0.1% |
| RWF | Maidstone and Tunbridge Wells | 738,831 | 35,784 | 4.8% | 699,589 | 35,781 | 5.1% | 94.7% | 100.0% | 0.3% |
| RWA | Hull University Teaching Hospitals | 736,942 | 60,094 | 8.2% | 735,068 | 59,839 | 8.1% | 99.7% | 99.6% | 0.0% |
| RQ8 | Mid Essex Hospital Services | 719,439 | 47,250 | 6.6% | 706,281 | 47,180 | 6.7% | 98.2% | 99.9% | 0.1% |
| RXR | East Lancashire Hospitals | 681,888 | 59,556 | 8.7% | 675,621 | 59,462 | 8.8% | 99.1% | 99.8% | 0.1% |
