# Supplementary material for: Characterising the nationwide burden and predictors of unkept outpatient appointments in the National Health Service in England: A cohort study using a machine learning approach
Source: PLoS Med. 2021 Oct 12;18(10):e1003783. doi: 10.1371/journal.pmed.1003783 (PMC8509877; doi:10.1371/journal.pmed.1003783)
Supplement: S3 Table — (DOCX) [file pmed.1003783.s003.docx]

S3 Table. Unkept appointment rates and model metrics for Imperial 2017-18 by specialty

Filtered for specialities with at least 10,000 appointments in 2016-17 (after data cleaning).

| **Speciality Name** | **Appointments** | **Unkept** | **Unkept %** | **Sensitivity** | **PPV** | **LR** | **AUROC** |
| --- | --- | --- | --- | --- | --- | --- | --- |
| ANTICOAGULANT SERVICE | 11990 | 1142 | 10% | 0.42 | 0.40 | 6.28 | 0.79 |
| PAEDIATRICS | 15474 | 1389 | 9% | 0.38 | 0.34 | 5.29 | 0.83 |
| NEPHROLOGY | 54839 | 5354 | 10% | 0.35 | 0.34 | 4.82 | 0.76 |
| COLORECTAL SURGERY | 16427 | 1709 | 10% | 0.35 | 0.35 | 4.71 | 0.75 |
| VASCULAR SURGERY | 12666 | 1772 | 14% | 0.31 | 0.43 | 4.70 | 0.72 |
| DIABETIC MEDICINE | 12182 | 1929 | 16% | 0.29 | 0.47 | 4.62 | 0.74 |
| BREAST SURGERY | 16838 | 1428 | 8% | 0.35 | 0.29 | 4.48 | 0.72 |
| CLINICAL HAEMATOLOGY | 26520 | 2658 | 10% | 0.33 | 0.33 | 4.45 | 0.73 |
| RESPIRATORY MEDICINE | 29390 | 4120 | 14% | 0.30 | 0.42 | 4.38 | 0.74 |
| ENDOCRINOLOGY | 26229 | 3700 | 14% | 0.30 | 0.42 | 4.41 | 0.71 |
| PLASTIC SURGERY | 11742 | 1091 | 9% | 0.32 | 0.30 | 4.12 | 0.74 |
| HEPATOLOGY | 14731 | 2494 | 17% | 0.28 | 0.46 | 4.10 | 0.74 |
| CARDIOLOGY | 43113 | 5001 | 12% | 0.30 | 0.35 | 4.04 | 0.73 |
| UROLOGY | 27746 | 2760 | 10% | 0.31 | 0.31 | 4.00 | 0.72 |
| GASTROENTEROLOGY | 22377 | 3198 | 14% | 0.28 | 0.40 | 3.97 | 0.71 |
| GENERAL SURGERY | 14137 | 1972 | 14% | 0.28 | 0.39 | 3.97 | 0.71 |
| DERMATOLOGY | 41267 | 5084 | 12% | 0.28 | 0.35 | 3.83 | 0.72 |
| RHEUMATOLOGY | 18182 | 2568 | 14% | 0.27 | 0.39 | 3.83 | 0.70 |
| GYNAECOLOGY | 62526 | 5508 | 9% | 0.31 | 0.27 | 3.82 | 0.72 |
| NEUROLOGY | 32833 | 4510 | 14% | 0.27 | 0.37 | 3.77 | 0.71 |
| NEUROSURGERY | 15302 | 1709 | 11% | 0.29 | 0.32 | 3.76 | 0.70 |
| OPHTHALMOLOGY | 74442 | 11188 | 15% | 0.26 | 0.39 | 3.63 | 0.71 |
| EAR NOSE THROAT | 34820 | 5109 | 15% | 0.26 | 0.38 | 3.61 | 0.69 |
| TRAUMA & ORTHOPAEDICS | 44966 | 6460 | 14% | 0.25 | 0.37 | 3.45 | 0.69 |
| MEDICAL ONCOLOGY | 36466 | 2052 | 6% | 0.29 | 0.16 | 3.30 | 0.69 |
| PHYSIOTHERAPY | 41143 | 4240 | 10% | 0.27 | 0.27 | 3.27 | 0.71 |
| ANAESTHETICS | 15690 | 1180 | 8% | 0.26 | 0.20 | 2.99 | 0.67 |
| MIDWIFERY SERVICE | 117205 | 12359 | 11% | 0.24 | 0.25 | 2.88 | 0.68 |
| AUDIOLOGICAL MEDICINE | 20577 | 1647 | 8% | 0.24 | 0.19 | 2.67 | 0.67 |
