# Supplementary material for: Characterising the nationwide burden and predictors of unkept outpatient appointments in the National Health Service in England: A cohort study using a machine learning approach
Source: PLoS Med. 2021 Oct 12;18(10):e1003783. doi: 10.1371/journal.pmed.1003783 (PMC8509877; doi:10.1371/journal.pmed.1003783)
Supplement: S4 Table — Filtered for specialties with at least 10,000 appointments in 2016–2017 (after data cleaning). (DOCX) [file pmed.1003783.s004.docx]

S4 Table. Predictor importance for each specialty (Filtered for specialities with at least 10,000 appointments in 2016-17 (after data cleaning))

| Speciality Name | **Appointments Last 12 Months** | **Unkept Appointments Last 12 Months** | **Cancellations Last 12 Months** | **Days Since Last Appointment** | **Days Since Last Unkept Appointment** | **Days Since Last Cancellation** | **Age at appointment** | **IDAOPI Score** | **IDACI Score** | **IMD Score** | **Health Deprivation Score** | **Consultation** | **Lead Care Professional** | **Appointment Type** | **Sex** | **Referral Source** | **Weekday** |
| --- | --- | --- | --- | --- | --- | --- | --- | --- | --- | --- | --- | --- | --- | --- | --- | --- | --- |
| ANTICOAGULANT SERVICE | 5.8% | 48.0% | 2.3% | 3.7% | 8.2% | 2.7% | 6.5% | 1.2% | 1.4% | 1.0% | 1.4% | 1.6% | 5.7% | 0.0% | 0.3% | 8.0% | 2.1% |
| PAEDIATRICS | 12.2% | 7.1% | 0.4% | 11.2% | 26.3% | 2.1% | 2.1% | 3.8% | 3.8% | 2.9% | 5.0% | 1.3% | 8.1% | 3.2% | 0.3% | 7.3% | 2.8% |
| NEPHROLOGY | 18.8% | 18.2% | 0.7% | 9.1% | 18.2% | 2.0% | 5.5% | 2.2% | 1.8% | 1.0% | 3.4% | 1.8% | 7.9% | 1.7% | 0.3% | 4.8% | 2.5% |
| COLORECTAL SURGERY | 6.7% | 8.3% | 0.4% | 4.9% | 26.5% | 1.3% | 13.1% | 4.8% | 3.0% | 4.5% | 4.9% | 1.0% | 10.8% | 3.1% | 0.8% | 3.4% | 2.6% |
| VASCULAR SURGERY | 5.6% | 14.3% | 1.2% | 4.9% | 25.9% | 1.1% | 5.4% | 3.5% | 1.2% | 1.3% | 5.7% | 2.6% | 20.1% | 1.6% | 0.4% | 3.4% | 1.7% |
| DIABETIC MEDICINE | 12.3% | 15.1% | 0.6% | 9.5% | 19.3% | 1.4% | 6.7% | 1.5% | 1.3% | 0.9% | 5.2% | 0.8% | 14.8% | 4.5% | 0.9% | 4.0% | 1.3% |
| BREAST SURGERY | 5.7% | 7.5% | 0.3% | 7.0% | 37.1% | 1.4% | 10.6% | 4.3% | 3.0% | 1.8% | 4.9% | 1.4% | 6.5% | 4.0% | 0.0% | 2.8% | 1.6% |
| CLINICAL HAEMATOLOGY | 13.6% | 18.4% | 0.6% | 7.9% | 21.9% | 1.4% | 10.0% | 3.3% | 1.6% | 1.7% | 2.7% | 1.7% | 8.7% | 1.3% | 0.3% | 3.0% | 1.9% |
| RESPIRATORY MEDICINE | 9.9% | 12.5% | 0.5% | 7.0% | 26.0% | 1.4% | 9.5% | 5.2% | 3.2% | 3.1% | 4.7% | 1.6% | 7.5% | 1.4% | 0.3% | 4.9% | 1.3% |
| ENDOCRINOLOGY | 10.4% | 15.7% | 0.7% | 5.3% | 32.4% | 1.8% | 6.9% | 3.2% | 1.5% | 1.3% | 3.1% | 0.6% | 7.9% | 2.9% | 0.6% | 3.0% | 2.8% |
| PLASTIC SURGERY | 2.5% | 5.9% | 0.2% | 3.5% | 17.5% | 1.1% | 14.1% | 1.5% | 2.3% | 1.1% | 16.7% | 0.6% | 22.8% | 0.9% | 0.7% | 6.8% | 1.7% |
| HEPATOLOGY | 9.3% | 16.4% | 0.6% | 7.8% | 29.5% | 1.7% | 5.9% | 2.4% | 2.3% | 4.3% | 4.8% | 3.7% | 3.7% | 0.9% | 0.4% | 3.3% | 3.1% |
| CARDIOLOGY | 6.9% | 15.7% | 0.4% | 5.0% | 25.0% | 1.3% | 7.3% | 7.3% | 5.2% | 4.5% | 7.2% | 1.3% | 4.3% | 1.7% | 0.3% | 5.1% | 1.5% |
| UROLOGY | 4.8% | 11.0% | 0.6% | 4.2% | 29.3% | 1.6% | 17.4% | 4.5% | 2.8% | 2.2% | 5.2% | 0.5% | 9.6% | 1.3% | 0.2% | 3.0% | 1.8% |
| GASTROENTEROLOGY | 9.1% | 11.6% | 0.4% | 6.6% | 28.3% | 1.6% | 8.4% | 3.8% | 1.7% | 2.0% | 4.5% | 0.8% | 9.1% | 3.9% | 0.5% | 5.9% | 1.6% |
| GENERAL SURGERY | 5.8% | 12.1% | 0.8% | 6.5% | 29.7% | 2.3% | 11.5% | 4.9% | 3.3% | 2.1% | 4.2% | 0.8% | 4.8% | 2.3% | 0.7% | 5.6% | 2.8% |
| DERMATOLOGY | 5.7% | 11.0% | 0.6% | 14.8% | 24.7% | 2.6% | 12.5% | 3.9% | 2.5% | 2.5% | 3.5% | 1.1% | 7.6% | 1.7% | 0.5% | 2.4% | 2.5% |
| RHEUMATOLOGY | 9.7% | 18.0% | 1.0% | 4.9% | 26.0% | 2.1% | 11.0% | 3.3% | 2.2% | 1.7% | 2.6% | 0.9% | 9.2% | 1.6% | 0.8% | 2.7% | 2.5% |
| GYNAECOLOGY | 3.4% | 7.8% | 0.6% | 8.5% | 26.9% | 2.4% | 6.1% | 4.4% | 2.7% | 2.2% | 7.6% | 1.5% | 11.4% | 3.7% | 0.0% | 8.2% | 2.5% |
| NEUROLOGY | 6.6% | 9.7% | 0.7% | 4.7% | 23.8% | 1.8% | 9.2% | 3.9% | 3.3% | 2.7% | 5.0% | 3.1% | 13.0% | 3.0% | 0.6% | 6.3% | 2.4% |
| NEUROSURGERY | 9.4% | 13.8% | 2.1% | 6.8% | 24.2% | 2.3% | 6.5% | 3.8% | 1.7% | 1.0% | 5.9% | 2.5% | 6.2% | 0.8% | 0.8% | 8.1% | 4.1% |
| OPHTHALMOLOGY | 5.9% | 11.4% | 0.5% | 4.3% | 28.3% | 1.1% | 13.6% | 6.4% | 3.9% | 3.1% | 4.8% | 1.1% | 6.3% | 0.7% | 0.3% | 6.9% | 1.4% |
| EAR NOSE THROAT | 3.8% | 9.5% | 0.4% | 4.4% | 25.4% | 1.6% | 15.2% | 5.0% | 2.6% | 1.6% | 7.2% | 0.8% | 16.0% | 1.4% | 0.4% | 3.3% | 1.6% |
| TRAUMA & ORTHOPAEDICS | 4.1% | 6.0% | 0.2% | 5.2% | 31.6% | 1.0% | 16.0% | 4.1% | 3.4% | 2.5% | 4.5% | 1.4% | 8.2% | 2.6% | 1.1% | 7.1% | 1.0% |
| MEDICAL ONCOLOGY | 7.2% | 8.2% | 1.6% | 8.2% | 13.7% | 3.6% | 6.3% | 10.5% | 6.9% | 6.4% | 8.3% | 5.3% | 5.3% | 1.8% | 0.4% | 3.3% | 3.2% |
| PHYSIOTHERAPY | 6.8% | 8.8% | 0.9% | 7.4% | 22.5% | 3.0% | 15.9% | 4.1% | 4.1% | 3.1% | 5.2% | 1.5% | 4.6% | 1.3% | 0.5% | 8.2% | 1.9% |
| ANAESTHETICS | 4.5% | 7.6% | 0.7% | 5.3% | 34.8% | 2.3% | 14.0% | 6.1% | 2.5% | 1.8% | 3.2% | 0.2% | 3.8% | 2.8% | 0.8% | 6.5% | 3.3% |
| MIDWIFERY SERVICE | 5.3% | 8.6% | 0.6% | 4.4% | 32.1% | 3.5% | 2.4% | 7.8% | 2.8% | 2.5% | 6.2% | 1.8% | 7.7% | 1.5% | 0.0% | 8.3% | 4.5% |
| AUDIOLOGICAL MEDICINE | 2.9% | 11.1% | 0.6% | 2.3% | 13.2% | 1.4% | 29.0% | 11.9% | 3.5% | 2.5% | 4.3% | 0.3% | 7.3% | 0.5% | 0.3% | 7.7% | 1.3% |
