# Supplementary material for: Characterising the nationwide burden and predictors of unkept outpatient appointments in the National Health Service in England: A cohort study using a machine learning approach
Source: PLoS Med. 2021 Oct 12;18(10):e1003783. doi: 10.1371/journal.pmed.1003783 (PMC8509877; doi:10.1371/journal.pmed.1003783)
Supplement: S1 Text — Fig A: Risk ratio of non-attendance for patients with an unkept appointment in the past 12 months. (DOCX) [file pmed.1003783.s005.docx]

## **S1 Text.** Model Specification

‘Recency, Frequency and Monetary value’ (RFM) models are in wide use in the commercial world, where they are used to relate the recency, frequency and monetary value of previous customer purchases to predict their future purchasing behaviour. Emulating this approach we calculated the recency (in days) and frequency (over the past 12 months) of appointments, unkept appointments and cancellations, to use as predictors of future patient attendances. Each record in our data provides a snapshot of what we know about a patient on the specific day of a given appointment. By including recency and frequency in the modelling we can determine whether previous behaviours predict future behaviours.

HES data from April 2016-March 2017 was used to train the models, then data from April 2017-March 2018 was used for testing. There was no overlap between the target appointments in the two datasets, but there was some information shared between the 2016-17 and 2017-18 snapshots. If a given patient has an appointment in 2016, then another appointment in 2017, then the information contained in this snapshot will be different; for example, whether or not they attended their 2016 appointment will be reflected in the ‘recency’ and ‘frequency’ calculations in their 2017 snapshot.

To check that this mechanism was not biasing our modelling we calculated the risk ratio on a month-by-month basis for patients with a previous unkept appointment in the last 12 months, versus patients with none, and we found that the risk ratio calculated on a month-by-month basis did not change substantially (averaging 2.76 RR), even though April 2017 has an 11 month overlap with 2016-17 data, while February 2018 has a 1 month overlap.

**Fig A. Risk ratio of non-attendance for patients with an unkept appointment in past 12 months**


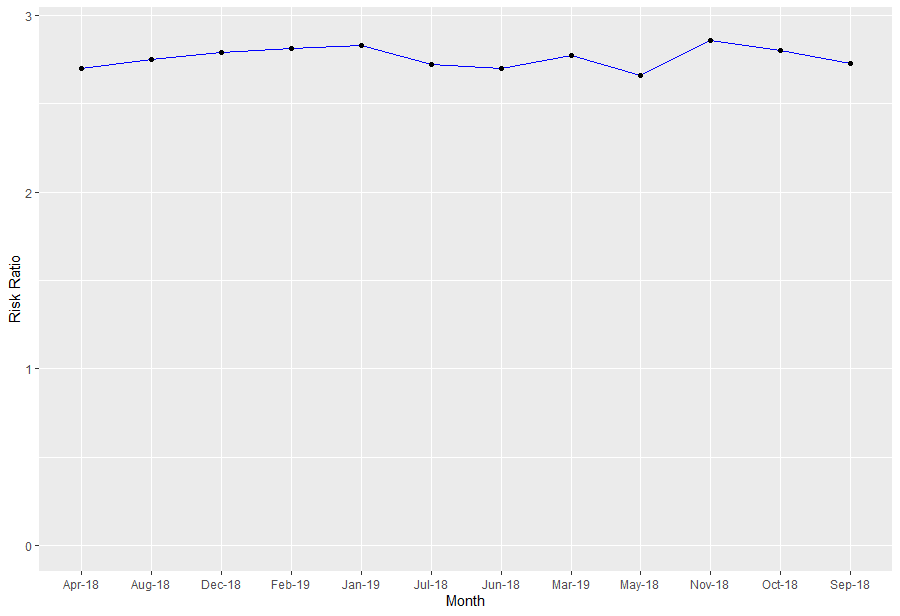


To train the gradient boosting machines we used the R package ‘gbm’, which uses Friedman’s stochastic gradient boosting algorithm [38,39]. The model hyperparameters were chosen during an initial exploratory analysis using a grid search, examining performance for a range of possible values, using cross validation with 10 folds, applied to 2016-17 data. For example, interaction depths between 3 and 8 were tested.

Based on the findings of the initial exploratory analysis, a standardised approach was subsequently taken for each of the specialism-specific models. A Bernoulli loss function was used to train models with the following hyperparameters: a maximum of 10,000 trees, with an interaction depth of 4, a bag fraction of 0.25, a minimum of 50 observations per node, and a shrinkage parameter (learning rate) of 0.001. Given the large sample size used for each model, and the fact that at least 50 observations are needed per node in the model, it is unlikely that the models would have been overfitted or biased by the same patients (with different appointments) appearing in each dataset, but if we were to rerun this analysis, we would split patients into different training and testing datasets.

The relative importance of the predictors is calculated by traversing each tree, recording how much mean square error is changed every time the predictor is used for splitting, then averaging the reduction across all the trees in the GBM. This value is then normalised, with the output mean, rank average and variation in importance displayed in Table 2.
