# Supplementary material for: Characterising the nationwide burden and predictors of unkept outpatient appointments in the National Health Service in England: A cohort study using a machine learning approach
Source: PLoS Med. 2021 Oct 12;18(10):e1003783. doi: 10.1371/journal.pmed.1003783 (PMC8509877; doi:10.1371/journal.pmed.1003783)
Supplement: Attachment — Submitted filename: PlosMed Response letter v4.docx [file pmed.1003783.s006.docx]

**Hutan Ashrafian**

Institute of Global Health Innovation Imperial College London

London

United Kingdom

Email: [h.ashrafian@imperial.ac.uk](about:blank)

Tel: +447799871597

27 January 2021

**Dr Clare Stone**

**PLOS Medicine**

Dear Dr Stone,

We wish to submit our revised manuscript entitled, ‘Characterising and reducing the burden of unkept outpatient appointments in the NHS through a national data-driven machine learning approach: a retrospective cohort study’. We thank you and your editorial team for your comments on our work, and we have amended our manuscript accordingly. I also attach a written response to the reviewers’ comments below, as well as a version of the main document in which these changes have been highlighted. Our responses are written in red and italicised below.

We look forward to your review of this manuscript.

Yours sincerely,

Mr Hutan Ashrafian BSc (Hons), MRCS, PhD, MBA

Chief Scientific Advisor, Institute of Global Health Innovation

Honorary Senior Clinical Fellow

Imperial College London

St Mary’s Hospital

Requests from the editors:

*Please structure your abstract using the PLOS Medicine headings (Background, Methods and Findings, Conclusions). We'd suggest including in the last sentence of the Abstract Methods and Findings section a note about any key limitation(s) of the study's methodology. Potentially this might include noting (this factor could also be included in the main Discussion section if the authors agree it is relevant) that independent validation in a separate dataset was not done for the predictors that emerged from the current machine learning study.

*In line with your guidelines, we have amended the abstract and added key limitations at the end. Lines: 45-48*

*At this stage, we ask that you include a short, non-technical Author Summary of your research to make findings accessible to a wide audience that includes both scientists and non-scientists. The Author Summary should immediately follow the Abstract in your revised manuscript. This text is subject to editorial change and should be distinct from the scientific abstract. Please see our author guidelines for more information: [https://journals.plos.org/plosmedicine/s/revising-your-manuscript#loc-author-summary](about:blank#loc-author-summary)

*We have added a non-technical summary, which follows the abstract. Lines 50-56*

*Please reformat the citation style into PLOS Medicine's format (should be straight forward if using referencing software) - this should use callouts formatted as sequential numerals in square brackets (not superscript).

*We have reformatted the citations as per your guidelines.*

*Please clarify if the analytical approach followed here corresponded to one laid out in a prospectively developed protocol or analysis plan? We'd ask the authors state this (either way) early in the Methods section.

a) If a prospective analysis plan (from your funding proposal, IRB or other ethics committee submission, study protocol, or other planning document written before analyzing the data) was used in designing the study, please include the relevant prospectively written document with your revised manuscript as a Supporting Information file to be published alongside your study, and cite it in the Methods section. A legend for this file should be included at the end of your manuscript.

b) If no such document exists, please make sure that the Methods section transparently describes when analyses were planned, and when/why any data-driven changes to analyses took place.

c) In either case, changes in the analysis-- including those made in response to peer review comments-- should be identified as such in the Methods section of the paper, with rationale.

*At the initial assessment process, the study was approved and deemed to be a service evaluation by NHS Digital.*

*We confirm all relevant ethical guidelines have been followed, and any necessary IRB and/or ethics committee approvals have been obtained. This secondary data analysis does not need IRB approval. All necessary patient/participant consent has been obtained and the appropriate institutional forms have been archived.*

*The editors would suggest referring to an appropriate reporting guideline to support reporting of the study, and one that may be appropriate is the RECORD guideline ([https://www.equator-network.org/reporting-guidelines/record/](about:blank)), developed for reporting of observational routinely-collected data. If the authors agree this is appropriate please enclose the completed RECORD checklist as supporting information with the revised paper.

*We have attached the RECORD checklist with the submission*

*We'd suggest including a descriptor of the study design as part of the manuscript title (normally this would be in the subtitle, after a colon - with the first part of the title including the study question/objective).

*As per your guidelines, we have added this to the title.*

*In the Introduction, the authors summarise prior evidence (in the section beginning "Reasons for unkept appointments often include...") but it would be good to then set out the unanswered question driving the current analysis, ie give the reader some idea of the uncertainty around prior research on unkept appointments and therefore what remains to be understood.

*We have now commented on previous studies in the background. Lines: 84-87*

-----------------------------------------------------------

Comments from the reviewers:

Reviewer #1:

"Characterising and reducing the burden of unkept outpatient appointments in the NHS through a national data-driven machine learning approach" studies the rate of outpatient clinic appointments within the National Health Service (NHS) of England, and employs machine learning models (in particular, gradient boosting machines; GBMs) to predict future missed appointments ("Did Not Attends"; DNAs), from available predictor variables (e.g. past appointments, unkept/cancelled appointments, etc - see Table 1 & Appendix 3). Accurate prediction of DNAs holds out the promise of substantial cost savings, since each (missed) appointment costs £120 to the NHS, for an annual wastage of about £1 billion.

Overall, this study raises the potential for both improving cost savings and patient health outcomes, through relatively easily-implemented means. The scale and scope of the HES (Hospital Episode Statistics) Outpatient Data used (containing close to 100 million appointments, and over 100 treatment specialties) is also a particular strength of the evaluation. However, there appear to remain a number of fairly significant issues with the current presentation, that might be addressed:

1. The first two citations supporting the costs of missed appointments to the NHS and the US healthcare system are to webpages. Might there be any alternative peer-reviewed publications that support these figures?

*In line with the above recommendation, we have additionally added a peer reviewed source:*

*Aggarwal A, Davies J, Sullivan R. “Nudge” and the epidemic of missed appointments: Can behavioural policies provide a solution for missed appointments in the health service? J Heal Organ Manag. 2016;30: 558–564. doi:10.1108/JHOM-04-2015-0061*

*The English citation comes directly from NHS data who are responsible for the collection and analysis of outpatient data.*

2. The background discussion includes prior work describing prevalence of and reasons for missing appointments, but does not really cover the closest relevant topic (i.e. on using machine learning/statistical methods to predict future unkept appointments) in detail. There appears to be a fair number of such papers, e.g. "Deprivation, demography and missed scheduled appointments at an NHS primary dental care and training service", West et al., British Dental Journal 228 (98-102), 2020; "Modeling patient no-show history and predicting future outpatient appointment behavior in the Veterans Health Administration", Goffman et al., Military Medicine 182 (5-6), e1708-e1714, 2017, etc.

*We have now added some references relating to others prediction models. Lines: 84-87*

3. The major methodological concern would be with the separation between the training dataset and the validation dataset. In particular, it is stated that "Appointments from April 2016 to March 2017 were used to train the models. The number of appointments, unkept appointments, and cancellations over the previous 12 months was also included. The models were tested using ICHT-specific data from April 2017 to March 2018. Again, including the number of appointments, unkept appointments and cancellations from the previous 12 months... HES data from 2016-17 was used to train the models, then (HES Outpatient data) data from 2017-18 was used for validation."

This would appear to imply that some of the information might be shared between the training and validation datasets. For example, for a given patient, an appointment in March 2017 (with accompanying 12-month prior data) would be employed as training data. However, it would seem that an appointment in say April 2017 by the same patient would then be used in validation, despite it likely sharing similar 12-month prior data (due to eleven of those months overlapping). The conventional arrangement then would then generally be to either stratify by patient, or at the trust level.

The authors might therefore comment on whether such a separation between the training and validation data was achieved, moreover since there might be come confusion as to whether the models were tested only on ICHT-specific data, or the full HES Outpatient data. In general, exactly what models were trained and validated on what data might be more systematically organized (e.g. a "composite prediction (GBM?) model" is suddenly mentioned in the Results section, but apparently not in the previous Methods section, while the GBM Model is only described in a later section after the Results section)

*In order to provide a more in-depth explanation to the reviewer’s comment, the model specification details have been added as an additional appendix to the paper. Please see appendix 4.*

*Recency, Frequency and Monetary value’ (RFM) models are in wide use in the commercial world, where they are used to relate the recency, frequency and monetary value of previous customer purchases to predict their future purchasing behaviour. Emulating this approach we calculated the recency (in days) and frequency (over the past 12 months) of appointments, unkept appointments and cancellations, to use as predictors of future patient attendances. Each record in our data provides a snapshot of what we know about a patient on the specific day of a given appointment. By including recency and frequency in the modelling we can determine whether previous behaviours predict future behaviours.*

*HES data from April 2016-March 2017 was used to train the models, then data from April 2017-March 2018 was used for validation. There was no overlap between the target appointments in the two datasets, but there was some information shared between the 2016-17 and 2017-18 snapshots. If a given patient has an appointment in 2016, then another appointment in 2017, then the information contained in this snapshot will be different; for example, whether or not they attended their 2016 appointment will be reflected in the ‘recency’ and ‘frequency’ calculations in their 2017 snapshot.*

*To check that this mechanism was not biasing our modelling we calculated the risk ratio on a month-by-month basis for patients with a previous unkept appointment in the last 12 months, versus patients with none, and we found that the risk ratio calculated on a month-by-month basis did not change substantially (averaging 2.76 RR), even though April 2017 has an 11 month overlap with 2016-17 data, while February 2018 has a 1 month overlap.*

***Figure 3. Risk ratio of non-attendance for patients with an unkept appointment in past 12 months***

*To train the gradient boosting machines we used the R package ‘gbm’, which uses Friedman’s stochastic gradient boosting algorithm. [39,40] The model hyperparameters were chosen during an initial exploratory analysis using a grid search, examining performance for a range of possible values, using cross validation with 10 folds, applied to 2016-17 data. For example, interaction depths between 3 and 8 were tested.*

*Based on the findings of the initial exploratory analysis, a standardised approach was subsequently taken for each of the specialism-specific models. A Bernoulli loss function was used to train models with the following hyperparameters: a maximum of 10,000 trees, with an interaction depth of 4, a bag fraction of 0.25, a minimum of 50 observations per node, and a shrinkage parameter (learning rate) of 0.001.*

*The relative importance of the predictors is calculated by traversing each tree, recording how much mean square error is changed every time the predictor is used for splitting, then averaging the reduction across all the trees in the GBM. This value is then normalised, with the output mean, rank average and variation in importance displayed in Table 3.*

4. The exclusion of cancellations in advance from the analysis would seem to affect the applicability of the results to a real-life implementation, since the trained GBM model would possibly be applied to cases that are eventually cancelled (rather than kept/missed). The authors might wish to discuss whether such cancellations are prevalent, and as such the extent to which they might affect the utility of the models.

*The reviewers have raised a valid concern and in an ideal scenario we would know when the intervention is being applied, and filter cancellations accordingly. For example, if patients are called 3 days before an appointment, then we could include cancellations within 3 days of appointments (as they haven’t happened yet), while excluding all cancellations that happen before the phone call would occur. However, we do not have cancellation date in our data, so it was not possible to take this approach.*

*According to official statistics (*[*https://digital.nhs.uk/data-and-information/publications/statistical/hospital-outpatient-activity/2018-19*](about:blank) *) the unkept appointment rate was 6.4% and the cancellation rate was 7.1% in 2018-19. Appointments can be cancelled by patients or by the care provider, and cancellations may occur shortly before an appointment, or well in advance.*

*Please see lines: 342-349*

5. For the metrics reported under the Gradient Boosting Machine Model section, the Likelihood Ratio and PPV are dependant on patients who were "selected for an intervention". Is this selection based on the 10% sensitivity cut off, and if so, might different cut offs for different specialities be considered given prior knowledge (e.g. 6% unkept appointment rates for medical oncology, compared to 17% for hepatology, from Appendix 2)?

*The reviewers have made a relevant suggestion and in a live service we would have different cut-offs for different specialities, based on their resources and respective rates of unkept appointments. However, in the context of analysing many specialities our results were more interpretable if we used a uniform cut off. This is explained on lines 306-308*

6. There are few details about the GBMs that would allow for some reproducibility. In particular, how were parameters such as the number of trees/shrinkage parameters/bagging fraction chosen? Was there any parameter search involved for each trained model? How was the importance of the various predictors determined? Was there any normalization of the inputs? These details might be appropriately covered in the appendix.

*In order to provide a more in-depth explanation to the reviewer’s comment, the model specification details have been added as an additional appendix to the paper. Please see appendix 4*

*The model hyperparameters were chosen during an initial exploratory analysis using a grid search, examining performance for a range of possible values of these hyperparameters, using cross validation with 10 folds, applied to 2016-17 data. For example, interaction depths between 3 and 8 were tested.*

*Based on the findings of the initial exploratory analysis, a standardised approach was subsequently taken for each of the specialism-specific models. A Bernoulli loss function was used to train models with the following hyperparameters: a maximum of 10,000 trees, with an interaction depth of 4, a bag fraction of 0.25, a minimum of 50 observations per node, and a shrinkage parameter (learning rate) of 0.001.*

*The relative importance of the predictors is calculated by traversing each tree, recording how much mean square error is changed every time a specific predictor is used for splitting, then averaging the reduction across all the trees in the GBM. This value is then normalised, with the output mean, rank average and variation in importance displayed in Table 3.*

7. The description of "Model sensitivity" mentions that "This is the biggest difference the intervention could possibly make". It is not immediately clear as to what this means.

*This means that unkept appointments could be reduced by 33%. We have now clarified this in the manuscript. Lines: 143-144*

8. The motivation for using predictive models for targeted interventions might be more extensively analyzed in the Discussion section. In particular, some interventions such as SMS reminders would appear to be feasible for all patients, without the need for a predictive model.

*The reviewers have raised a relevant point, however we have added a section in the discussion explaining why a blanket approach may not be appropriate. Lines: 288-293*

9. There remain a small number of possible grammatical issues, e.g. "top 10% less likely" -> "top 10% least likely"; references to tables and appendices in the text are generally capitalized.

*We have addressed the grammatical issues.*

-----------------------------------------------------------

Reviewer #2:

This paper is an excellent piece of work. it clearly identifies the issues attributed to unkept appointments and states the research aims very clearly.

The statistical terms have been very well explained as was your methodology.

The tables clearly stated the results, particularly rates and numbers per location and specialisation very succinctly.

I have no criticisms to add of the paper, it is a well written and executed research paper.

-----------------------------------------------------------

Reviewer #3:

General Feedback- major aspects

Thank you for submitting this manuscript for consideration and I read it with interest as a clinician and researcher who works in this field.

I do not however have any expertise in machine learning and appreciated the description of key terms included.

It would be useful for the reader if you were to explain the change in language from missed to unkept; its not clear, because missed apts have not ever included cancelled apts in the data outputs.

*In order to be consistent, we are now using “unkept” throughout the text.*

The focus on good population coverage is welcomed and the clear description of the data being used, its benefits and drawbacks. You attempted as best as I was able to discern (not being a stats expert)  to account for the data recording variation with respect to missed/unkept appointments. However does this not also mean that some of the most stark data about missingness may have been contained in the top 10%?

*Yes,* *data with the most missingness would have been among the top 10%. This is now clarified on line: 331*

I note that mental health services are not included? In Scottish data they are. Could they all have been in the top 10% or are they not included in HES OP data? I think you need to explain why secondary care mental health services are not included in this data set. I'm making this point because the hospital activity data linked to the GP publshed data you cited does include mental health services and they were the highest generator of repeated missed apts in the Scottish data (currently under review for publication).

*Unfortunately, the psychiatric and paediatric psychiatry records included in HES are limited. However, but greater detail on these records is found in the Mental Health Services dataset (MHSDS). ICHT does not have a dedicated psychiatry department and utilises liaison psychiatry services from partner London trusts and therefore mental health services were not looked at in this study. We have acknowledged this, lines: 334-336*

I also think that you need to explain  in applied terms why exluding the top 10% may impact on the results (even if it may make sense from a machine learning perspective).

*We thank the reviewers for this point and have discussed it on lines: 337-342*

*Excluding only the trusts with the 10% highest rate of unkept appointments would potentially result in the model under-predicting unkept appointments. However, we also excluded the outliers with the 10% lowest rate of unkept appointments, and so there is likely to be some cancelling out. Additionally, excluding records which are likely to have lower data quality could improve the accuracy of the predictions (because there is more information for the trained algorithm to use, and because there would be less unrelated anomalies left in the cleaned data to distort results). On the other hand, trusts which were excluded would be less represented in the data, so the model might be less applicable to their particular circumstances.*

Is previous unkept appointments by specialty or any unkept appt, please clarify? Because one potential explanation for high missed appts is patient treatment burden, the patient has so many apts across multiple specialties that they cannot manage to keep them all, which may be why it is linked to cancelled apts too.

*Previous unkept appointments is calculated by specialty. Line: 121*

What evidence given the lack of population data on this topic means you can speculate about needs being met elsewhere (line 220 to 231)?

*We have removed this speculation from the discussion.*

Can you really speculate about rates of unkept appts by English regions when a significant contribution to the data differences may be about data recording and quality if missed appointments are not mandated to be returned? Could this be relevant at specially level too?

*Unfortunately, this is a limitation of our study. We have had to make the assumption that if the submission from a trust looks reasonable, then it is correct. We have acknowledged this in the discussion, lines: 321-324*

I'm uncomfortable with the amount of time spent on possible interventions in the manuscript (250-264)- as the most important finding from this research is that  previous missed apts are the strongest predictor of future missingness. As far as I am aware no large or medium scale work has yet evaluated interventions that may work that focus on a history of previous missingness. The evaluations done at any scale do not distinguish between patients who miss one OP apt and those who miss many (and hence probably why studies often contradict each other). This focus on patients at high risk of a pattern of missingness and what works is what is needed- that's my reading of what this important study tells us. And we cannot speculate on what works until research focussed on patients with patterns of high missingness is conducted.

*We have reduced the part of the discussion to minimise speculation. Lines: 272-287*

As you are probably aware the evidence that underpins the economic cost per missed apts in the UK is flimsy at best (line 49) and it could be argued have some positive cost benefit (clinicians doing catch up letters etc), so I would suggest including 'estimate' at the very least in your statement about this.

Linked to this would an important recommendation from this work not be that NHS Digital should mandate returning recording of all missed/unkept appointments in line with the need to return all attended ones too? Given that you present a strong case for attention to be paid to this issue.

*Whilst the reviewers have made a reasonable suggestion, NHS Digital cannot mandate this, however we have added the following statement:*

*We would recommend digital health policy makers mandating trusts to record and submit unkept appointments, in addition to those attended, to avoid this issue for future related research. Lines 351-352.*

Minor aspects

Appointment age as a term is misleading- I had to read further down to confirm that it is the age of the patient and not the time elapsed since the appointment was scheduled.

*As “appointment age” was not clear, we have now re-worded this to “age at appointment”.*
