# Supplementary material for: Characterising the nationwide burden and predictors of unkept outpatient appointments in the National Health Service in England: A cohort study using a machine learning approach
Source: PLoS Med. 2021 Oct 12;18(10):e1003783. doi: 10.1371/journal.pmed.1003783 (PMC8509877; doi:10.1371/journal.pmed.1003783)
Supplement: Attachment — Submitted filename: PlosMed Response letter v5.docx [file pmed.1003783.s007.docx]

**Hutan Ashrafian**

Institute of Global Health Innovation Imperial College London

London

United Kingdom

Email: [h.ashrafian@imperial.ac.uk](about:blank)

Tel: +447799871597

14 August 2021

**Dr Clare Stone**

**PLOS Medicine**

Dear Dr Stone,

We wish to submit our revised manuscript entitled, ‘Characterising the nationwide burden and predictors of unkept outpatient appointments in the NHS in England: A cohort study using a machine-learning approach’. We thank you and your editorial team for your comments on our work, and we have amended our manuscript accordingly. I also attach a written response to the reviewers’ comments below, as well as a version of the main document in which these changes have been highlighted. Our responses are written in red and italicised below.

We look forward to your review of this manuscript.

Yours sincerely,

Mr Hutan Ashrafian BSc (Hons), MRCS, PhD, MBA

Chief Scientific Advisor, Institute of Global Health Innovation

Honorary Senior Clinical Fellow

Imperial College London

St Mary’s Hospital

Requests from Editors:

Please adapt the title to better match PLOS Medicine style. We suggest: "Characterising the nationwide burden and predictors of unkept outpatient appointments in the NHS in England: A cohort study using a machine-learning approach".

*We have amended the title to the above suggestion*

At line 33, please make that "... Data ... were used ...".

*This error has been corrected*

At line 39 (abstract), we suggest quoting the estimated proportions contributed by the three predictors mentioned.

*We have now added the percentages: The most important predictors of unkept appointments included the recency (25%) and frequency (13%) of previous unkept appointments and age at appointment (10%).*

Please remove the subsection headed "Key limitations" in your abstract. This material should be located in a new final sentence in the "Methods and findings" subsection, which should begin "Study limitations include ..." or similar and should quote 2-3 of the study's main limitations.

*The study limitations have been added to the “Methods and findings subsection”. Please refer to lines 41-43*

Please remove the "non-technical summary" following the abstract and instead craft an accessible "Author Summary" section. You may find it help to consult one or two recent research papers in PLOS Medicine to get a sense of the preferred style.

*We have removed the non-technical summary and provided the following Author Summary (lines 49-57):*

*•	Unkept appointments cost the NHS £1 billion annually*

*•	We determined rates and examined the predictors of unkept outpatient clinic appointments across specialities*

*•	We found that those who had previously missed their appointment were most likely to miss another appointment*

*•	Considering this, have identified a cohort of patients for whom targeted interventions can be implemented, to reduce the number of unkept appointments to improve patient care and reduce costs to healthcare providers*

Please state early in the Methods section whether or not the study had a protocol or prespecified analysis plan.

*We have added the following to the methods section (line 139-140):*

*“This was a post hoc analysis, based on observational data, and did not have a prespecified study plan.”*

Please refer to the attached RECORD checklist in the Methods section ("See S1_RECORD_Checklist" or similar).

*We have now referred to this in the methods section, line 158*

Please restructure the start of the Discussion section, as the first paragraph should summarize the study's findings (it appears that this would be achieved if the first two current paragraphs of this section were amalgamated).

*As per your suggestion, the two paragraphs have been amalgamated, lines: 233-239*

Please adapt reference call-outs to precede punctuation throughout the text (e.g., "... up to 6 months [5,6].").

*These have been amended.*

Please remove the information on study funding and competing interests form the end of the main text. This information will appear in the article metadata in the event of publication, via entries in the submission form.

*We have removed this section.*

Noting reference 4, please ensure that all citations have full access details.

*We have now provided an appropriate reference with full access details*

*Carreras-García D, Delgado-Gómez D, Baca-García E, Artés-Rodriguez A. A Probabilistic Patient Scheduling Model with Time Variable Slots. Comput Math Methods Med. 2020;2020: 9727096. doi:10.1155/2020/9727096*

Please use the journal name abbreviation "PLoS ONE" in the reference list.

*This has been rectified*

Please remove all iterations of "[Internet]" from the reference list.

*“Internet” has been removed*

Comments from Reviewers:

*** Reviewer #1:

We thank the authors for addressing our previous concerns, particularly for the additional Appendix 4 detailing the model specification, and the analysis suggesting that the data leakage did not significantly bias the findings (as illustrated in Figure 3). While the information on RFM models are noted, in principle it would be more ideal to have the training and validation datasets not sharing the same patients. This caveat might be briefly acknowledged if thought appropriate.

*We have added the following to appendix 4:*

*“Given the large sample size used for each model, and the fact that at least 50 observations are needed per node in the model, it is unlikely that the models would have been overfitted or biased by the same patients (with different appointments) appearing in each dataset, but if we were to rerun this analysis, we would split patients into different training and testing datasets.”*

*** Reviewer #3:

Thank you for addressing my feedback comprehensively.

There are some minor typo errors- line 46, 87, 170.

*These have been corrected*
