# Supplementary material for: Characterising the nationwide burden and predictors of unkept outpatient appointments in the National Health Service in England: A cohort study using a machine learning approach
Source: PLoS Med. 2021 Oct 12;18(10):e1003783. doi: 10.1371/journal.pmed.1003783 (PMC8509877; doi:10.1371/journal.pmed.1003783)
Supplement: Attachment — Submitted filename: PlosMed Response letter v7.docx [file pmed.1003783.s008.docx]

**Hutan Ashrafian**

Institute of Global Health Innovation Imperial College London

London

United Kingdom

Email: [h.ashrafian@imperial.ac.uk](about:blank)

Tel: +447799871597

24 August 2021

**Dr Richard Turner**

**PLOS Medicine**

Dear Dr Tuner,

We wish to submit our revised manuscript entitled, ‘Characterising the nationwide burden and predictors of unkept outpatient appointments in the NHS in England: A cohort study using a machine-learning approach’. We thank you and your editorial team for your comments on our work, and we have amended our manuscript accordingly. I also attach a written response to the reviewers’ comments below, as well as a version of the main document in which these changes have been highlighted. Our responses are written in red and italicised below.

We look forward to your review of this manuscript.

Yours sincerely,

Mr Hutan Ashrafian BSc (Hons), MRCS, PhD, MBA

Chief Scientific Advisor, Institute of Global Health Innovation

Honorary Senior Clinical Fellow

Imperial College London

St Mary’s Hospital

Requests from Editors:

At line 26, please make that "appointments cost".

*This has been amended*

At line 41 in the abstract, immediately prior to the sentence summarizing study limitations, we feel that an additional sentence or two should be added to summarize the inferences from the GBM work beginning at line 205 in the Results. The information about sensitivity may be the most intuitive finding to report in the abstract.

*We have added the following to the abstract:*

*“A sensitivity of 0.287 was calculated overall for specialties with at least 10,000 appointments in 2016-17 (after data cleaning). This suggests that 28.7% of patients who do miss their appointment would be successfully targeted if the top 10% least likely to attend received an intervention. As a result, an intervention targeting the top 10% of likely non-attenders, in the full population of patients, would be able to capture 28.7% of unkept appointments if successful.” Lines: 41-45*

At line 44, please make that "... appointments remain ..." or similar.

*This has been amended*

Please revisit the "Author summary", which should consist of three subsections with the following headings, each comprising 3-4 short points (in turn of 1-2 short sentences each):

*We have amended the author summary to:*

*“Why was the study done?*

*•	Unkept appointments cost the NHS £1 billion annually*

*What did the researchers do and find?*

*•	We determined rates and examined the predictors of unkept outpatient clinic appointments across specialities*

*•	We found that those who had previously missed their appointment were most likely to miss another appointment*

*What do these findings mean?*

*•	Considering this, we have identified a cohort of patients for whom targeted interventions can be implemented, to reduce the number of unkept appointments to improve patient care and reduce costs to healthcare providers”*

Please use the active voice (e.g., "We investigated ...) in at least one point.

*We have amended the methods section to utilise the active voice rather than the passive.*

At line 64, we suggest "impacts".

*This has been amended*

At line 66, we suggest "... missed more than 2 appointments".

*This has been amended*

At line 86, please make that "... carried out in a UK setting ...".

*This has been amended*

At line 90, please make that "potential utility".

*This has been amended*

At lines 101 and 207, please make that "[data] were used ...".

*These have been amended*

At line 119, should that be "... appointments and unkept appointments ..."?

*The editor is correct, and as such, this has been amended*

At line 187, for example, please avoid using italics for emphasis.

*This has been amended*

At line 260, please make that "existing literature".

*This has been amended*

At line 277, please make that "have been shown".

*This has been amended*

At line 278, please revisit the wording - perhaps "... the present findings will allow us to go a step farther ..." is intended?

*The editor is correct, and as such, this has been amended*

At line 352, please make that "break down" (two words).

*This has been amended*

At line 359, please remove the information on competing interests, which will appear in the article metadata via entries in the submission form.

*This has been removed*
